# Supplementary material for: Estimated impact of the pneumococcal conjugate vaccine on pneumonia mortality in South Africa, 1999 through 2016: An ecological modelling study
Source: PLoS Med. 2021 Feb 16;18(2):e1003537. doi: 10.1371/journal.pmed.1003537 (PMC7924778; doi:10.1371/journal.pmed.1003537)
Supplement: S1 Text — (PDF) [file pmed.1003537.s002.pdf]

S2. Analysis plan (25-04-2018)

Estimated impact of the pneumococcal conjugate vaccine on pneumonia mortality in South Africa, 1999 through 2016: an ecological modelling study. Kleynhans et. al.

<u>Background</u>

The pneumococcal conjugate vaccine (PCV) was introduced into the South African routine immunisation programme in 2009. By 2012, invasive pneumococcal disease (IPD) rates in children aged <2 years had declined by 69%. Rates of IPD in adults aged 25-44 years had also declined by 34%, likely as a result of indirect effect.

The pneumococcus is an important cause of pneumonia deaths, estimated to cause XX (?33%-80%) of all pneumonia deaths globally. (Ref 43,44,53 get from Rudan) Pneumococcus is estimated to cause 50-60% of bacterial meningitis cases in children <5 years and 10% of all meningitis in adults aged ≥18 years in South Africa. (Meiring unpub, Britz submitted) The clinical trial of PCV efficacy in the Gambia found an 16% reduction in all-cause mortality in a rural African setting. Other studies have estimated that pneumococcus causes 11% of all deaths in children aged <5 years globally.[10] The relative contribution of pneumococcus and other bacteria to pneumonia mortality may be higher in high mortality settings.(Ref) Quantifying the impact of PCV on mortality is important in order to understand the total impact of the vaccine.

Over the past decade there have been substantial improvements in the availability and effectiveness of interventions to prevent mother-to-child transmission of HIV (PMTCT), leading to marked reductions in the number of HIV-infected infants born each year in South Africa. In addition, there has been marked expansion of the availability of HIV antiretroviral treatment (ART) for HIV-infected individuals. These interventions have had a substantial impact on all-cause and pneumonia mortality in South Africa. (Ref Johnson AIDS 2013)

HIV-infected individuals have approximately 20-40 times increased risk of developing invasive pneumococcal disease (IPD). (Ref) [11] HIV is the most important risk factor for IPD in South Africa, with 30% of children aged <5 years and >80% of individuals aged ≥5 years with IPD having documented HIV infection.(Ref)[11]

We aimed to estimate the impact of PCV on respiratory and meningitis mortality in South African individuals of all ages controlling for changes in HIV prevention and treatment from 1998-2013.

Objectives

1. Estimate the impact of PCV on pneumonia and meningitis deaths in South Africa by age group using national mortality data from 1999-2015 (pre-PCV 1999-2008, post PCV 2010-2015).

Methods

- Plan to use weinberger synthetic controls approach
- To do this need to:
    - Download latest available death data
    - Create large merged death data file
    - Format data correctly for Weinberger analysis
        - ICD codes for outcome and synthetic controls – Need to really drill down into these codes and decide what to include and what not
        - Dates – daily weekly etc

        - Age groups – to decide – <2, 2-4, 5-14, 15-24, 25-44, 45-64, >64
    - Download R files and load R
    - Decide which controls are appropriate to include as potential synthetic controls – impacted by HIV but not rota vaccine – can try to train on the pre PCV, post ART years to identify good predictors of trends
    - Consider whether to include flu and RSV, think about whether need to deal with pandemic
    - Consider whether to include specific HIV covariates eg ART use etc in model or will the synthetic controls do the work?
    - Evaluate data for obvious changes in coding practices
    - Decide years to use for impact evaluation - ? final 2 years of available data
    - Initial attempt to use code and troubleshoot
