# Supplementary material for: Estimated impact of the pneumococcal conjugate vaccine on pneumonia mortality in South Africa, 1999 through 2016: An ecological modelling study
Source: PLoS Med. 2021 Feb 16;18(2):e1003537. doi: 10.1371/journal.pmed.1003537 (PMC7924778; doi:10.1371/journal.pmed.1003537)
Supplement: S2 Text — (PDF) [file pmed.1003537.s003.pdf]

S3. Detailed methods

Estimated impact of the pneumococcal conjugate vaccine on pneumonia mortality in South Africa, 1999 through 2016: an ecological modelling study. Kleynhans et. al.

Estimating the impact of PCV on all-cause pneumonia mortality

Individual-level mortality data were grouped according to ICD-10 chapter, month of death, and age group. Age strata in children were selected to assess the impact of PCV, based on year of birth, on those eligible for routine vaccination (1 month to 4 years), those who could possibly have been vaccinated in catch-up campaigns (born before February 2009) or routine vaccine (born February 2009 – 2011, 5-7 years) and those not eligible for vaccination (born before 2009, 8-18 years) in 2016 the final year of data included in the analysis. In adults, the age stratum with highest HIV prevalence (19-39 years) was separated from other age groups. Infants aged <1 month were excluded from the analysis since pneumonia may not present with typical symptoms in this age group but rather sepsis, which was not included in our outcome [1]. The percentage of deaths related to pneumonia based on using the P23 (congenital pneumonia) ICD-10 code was only 6% of total deaths for this age group. Where date of death (49/9 324 638, <0.001%) or date of birth/age was missing (36,144/9 324 638, 0.38%) deaths were not included in the analysis. Cause of death was complete for records.

The synthetic control analysis rests on three major assumptions: 1) that the association between pneumonia deaths and the control causes of death is stable over time (e.g., not influenced by other disease-specific interventions for example vaccines, 2) that PCV does not affect the control conditions, and 3) that the control conditions share important causal factors with pneumonia deaths and thus are able to effectively adjust for trends unrelated to PCV [2]. We therefore excluded deaths with ICD-10 codes that could also have been affected by PCV, i.e., those related to pneumonia, septicemia, bacterial infections of unspecified site, bacterial meningitis, conjunctivitis or otitis media in any of the six cause of

death fields, as well as those related to infectious diarrheal disease which may have been influenced by the introduction of the rotavirus vaccine in 2009. Details of excluded codes are shown in Table S1. We log-transformed all time series before using these in the analyses, and transformed observed and predicted values back to original scale before calculating rate ratios and cases prevented. This process was used to reduce the effects of epidemics on long-term trends and associations [2].

We constructed time series of the 38 ICD-10 groupings (i.e. controls, all log-transformed, Table S1) and then used Bayesian variable selection to determine their contribution to the synthetic control, weighting each group more or less based on how well it fit the pneumonia deaths time series during 1999-2008. Variables were selected using spike-and-slab priors with equal prior probability for inclusion for all covariates [3], and this probability was set so that the prior inclusion probability for each variable was $\pi = 0.5$ [2]. We then fitted a regression model (equation S1) to the monthly pre-vaccine number of pneumonia deaths (log-transformed) using a Bayesian structural time-series model, drawing 10 000 samples using Markov Chain Monte Carlo (MCMC). This allowed us to quantify uncertainty associated with variable selection and variation in the data. We used the bsts [4] and CausalImpact [3] packages in R for model fitting and formatting of output. This process generated an equation that described the relationship between the control variables and pneumonia deaths in the absence of the vaccine [2]. A composite control was generated by plugging in the observed values of the control diseases during the post-PCV period into this equation. The output from this model provided an estimate for the expected number of pneumonia deaths if the intervention had not been introduced for the post-PCV period (the counterfactual).

$$\log(\text{pneumonia cases}_t) = \beta_0 + \sum_k [c_k * l\{\text{month}_k = m(t)\}] + \sum\left[\text{binary\_inclusion\_indicator}_j * \text{coeff}_j * \log(\text{control\_time\_series}_{jt})\right]$$

[S1]

In this model, *Σ[binary_inclusion_indicator*$_j$**coeff*$_j$**log(control_time_series*$_{jt}$*)]* is the set of control time series, weighted by their inclusion indicators and regression coefficients. The

binary_inclusion_indicator$_j$ are independent Bernoulli (π)-distributed random variables that determine the presence or absence of a particular control series in the model (0: excluded; 1: included). Seasonality of the outcome was controlled for by 11 monthly dummy variables [2].

Sensitivity analyses

Prior to 2006 there was a systematic misclassification of ICD codes among children aged <1 year into the P code groups which are classified as 'Certain conditions originating in the perinatal period', although it might not have been a child who died within the first seven days of live [5]. For the purpose of this study, all P codes used in children aged >1 month were grouped with non-P codes where possible, as previously described [5]. For example, if a death was coded as P35-P39 (Infections specific to the perinatal period) it was included in the control group that included the A and B chapters (Certain infectious and parasitic diseases) and deaths with P23 in any of the cause of death fields were included as a pneumonia death. The grouping of codes is shown in Table S1.

Due to unknown HIV status at time of death, or the deliberate omission of HIV from the death certificate in fear of stigma, losing health insurance benefits, or confidentiality concerns, many HIV-related deaths have not been coded as B20-B24 [6]. Since many of these deaths were rather coded as infectious or parasitic diseases compiled in the A and B ICD chapters, as well as deficiency anemias housed in the D50-D89 chapters, the A20-A99 [7], B00-B99, and D50-D89 chapters were compiled into one control group to ensure sufficient control for the effect of HIV infection on mortality. To assess the effect of these reclassifications on the results, the model was repeated with no P code re-classification and without grouping D50-D89 with A10-B99.

It was unclear whether to include ICD-10 Chapter-10 XVIII (codes R00-R99; 'Symptoms, signs and abnormal clinical and laboratory findings, not elsewhere classified') in the analysis. This chapter contains ill-defined causes of death. Due to the non-specific nature of these

codes, some pneumonia deaths might have been classified as such, and by including this chapter as part of the controls, we could reduce the effect observed. However, there may be some HIV-related deaths included in these controls, and if excluded, may lead to insufficient control for changes in HIV-related deaths or coding biases. We elected to include this chapter as a control in the main analysis but conducted a sensitivity analysis where it was excluded.

We also performed a sensitivity analysis to identify whether the model results were sensitive to other controls selected and which years were included, by repeating the model and excluding different controls or years in each individual run. The group of controls including respiratory deaths J00-J99 and the group including other lower respiratory tract infection deaths J20-J22 (bronchitis and bronchiolitis) were removed respectively. We also removed the following years: 1999-2005 (excluding timeframe in which pneumonia mortality increased in South Africa, and abrupt shift in use of R codes in 1-11month group), 2009 (to assess influence of influenza pandemic) and 2015-2016 (reducing evaluation period by 2 years to assess signal for vaccine replacement), all in different analyses. We also aggregated the data by trimester (January – April, May – July and August – December) to reduce data points and assess for possible model overfitting.

To compare results from the synthetic control approach, we also performed an interrupted time series analysis to compare deaths before and after the introduction of the vaccine. The number of post-vaccine counterfactual deaths was based on the number of pre-vaccine period deaths adjusted for seasonal variations by using a month variable. Rate ratios were calculated by dividing the summed of post-vaccine deaths by the summed counterfactual deaths in the 2012-2016 period.

Exclusions from controls

Any ICD-10 codes relating to pneumonia or that could possibly relate to pneumococcal disease was not included as controls. These included septicaemia (A40, A41), bacterial infections of an unspecified site (A49), bacterial meningitis (G00, G03), conjunctivitis (H10), otitis media and other ear

infections (H66, H70, A74) and pneumonia (J12-J18). Furthermore, the rotavirus vaccine was introduced in South Africa in August 2009[8], in the same year as PCV. One of the assumptions of the synthetic control approach is that the controls used has not been subjected to a separate intervention [2], and therefore ICD-10 codes those relating to infectious diarrheal disease (A00-A09) were also excluded.

Other changes in the South Africa expanded programme on immunisation during the study period was the introduction of Haemophilus influenzae type b (Hib) vaccine in 1999, which would have also led to a reduction in pneumonia deaths prior to 2009 of which the synthetic control approach would have controlled for. The only other changes were swopping whole-cell pertussis vaccine to acellular vaccine in 2009, and opting for the hexavalent vaccine (polio, diphtheria, tetanus, pertussis, Hib) in 2015 by replacing the 5-in-1 vaccine and Hepatitis B vaccines [9]. We did not expect these changes to have an influence on our analysis.

References

1. McIntosh K. Community-Acquired Pneumonia in Children. N Engl J Med. 2002;346(6):429-37. doi: 10.1056/NEJMra011994. PubMed PMID: 11832532.

2. Bruhn CA, Hetterich S, Schuck-Paim C, Kurum E, Taylor RJ, Lustig R, et al. Estimating the population-level impact of vaccines using synthetic controls. Proc Natl Acad Sci U S A. 2017;114(7):1524-9. Epub 2017/02/06. doi: 10.1073/pnas.1612833114. PubMed PMID: 28154145; PubMed Central PMCID: PMCPMC5321019.

3. Brodersen KH, Gallusser F, Koehler J, Remy N, Scott SL. Inferring causal impact using Bayesian structural time-series models. Ann Appl Stat. 2015;9(1):247-74. doi: 10.1214/14-AOAS788.

4. Scott SL. bsts: Bayesian Structural Time Series. R package version 0.6.2. 2020 29 October]]. Available from: https://cran.r-project.org/web/packages/bsts/.

5. Pillay-Van Wyk V, Laubscher R, Msemburi W, Dorrington R, Groenewald P, Vos T, et al. Second South African National Burden of Disease Study: Data cleaning, validation and SA NBD List. Cape Town2014.

6. Birnbaum JK, Murray CJ, Lozano R. Exposing misclassified HIV/AIDS deaths in South Africa. Bull World Health Organ. 2011;89(4):278-85. Epub 2011/02/17. doi: 10.2471/BLT.11.086280. PubMed PMID: 21479092.

7. Groenewald P, Nannan N, Bourne D, Laubscher R, Bradshaw D. Identifying deaths from AIDS in South Africa. AIDS. 2005;19(2):193-201. Epub 2005/01/26. PubMed PMID: 15668545.

8. Seheri LM, Page NA, Mawela MP, Mphahlele MJ, Steele AD. Rotavirus vaccination within the South African Expanded Programme on Immunisation. Vaccine. 2012;30 Suppl 3:C14-20. Epub 2012/09/04. doi: 10.1016/j.vaccine.2012.04.018. PubMed PMID: 22939015.

9. Dlamini NR, Maja P. The Expanded Programme on Immunisation in South Africa: A story yet to be told. S Afr Med J. 2016;106(7):675-7. Epub 2016/07/08. doi: 10.7196/SAMJ.2016.v106i7.10956. PubMed PMID: 27384357.
