# Supplementary figures and images for: Estimated impact of the pneumococcal conjugate vaccine on pneumonia mortality in South Africa, 1999 through 2016: An ecological modelling study
Source: PLoS Med. 2021 Feb 16;18(2):e1003537. doi: 10.1371/journal.pmed.1003537 (PMC7924778; doi:10.1371/journal.pmed.1003537)

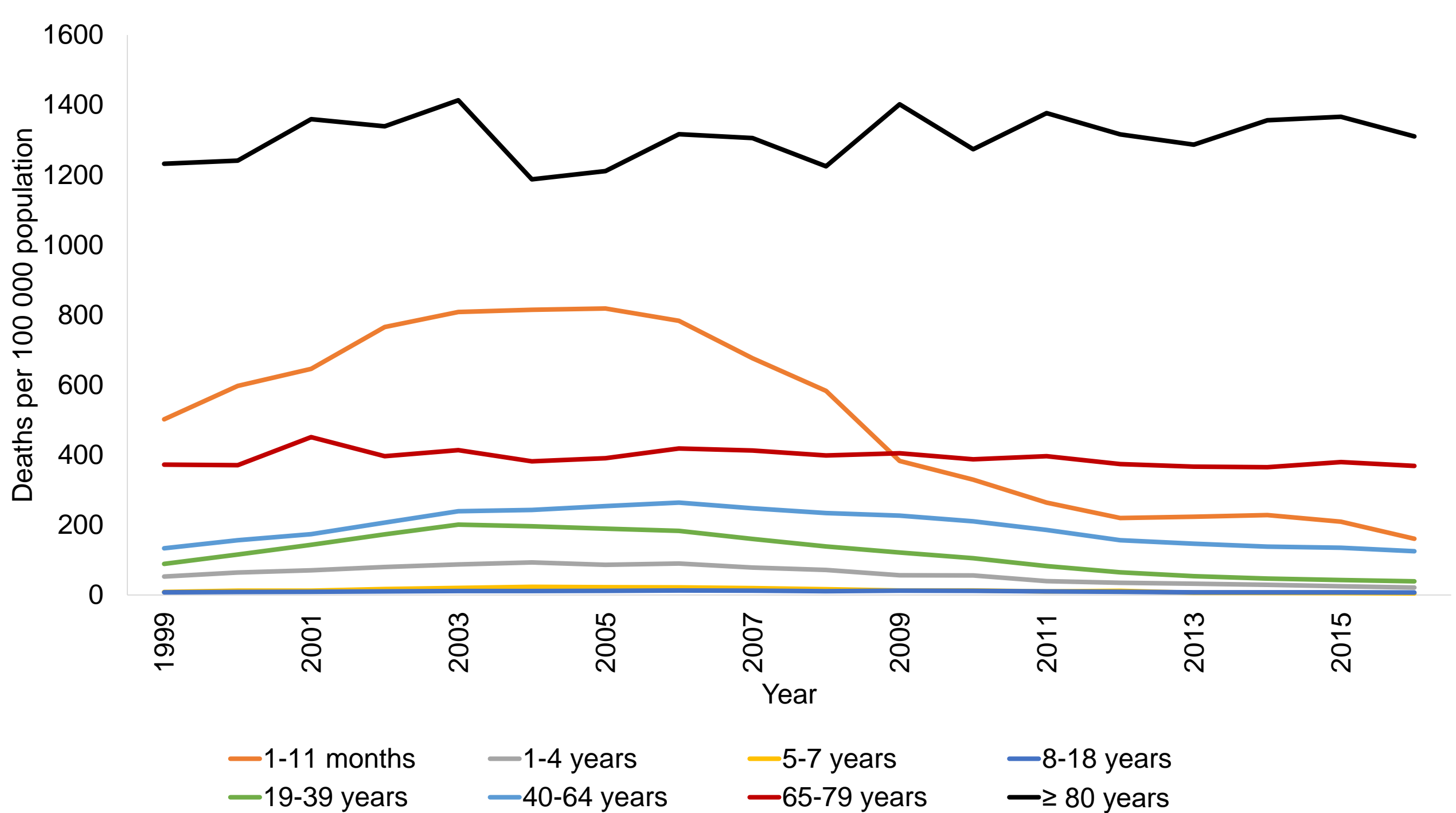

Supplement: S1 Fig — (PDF) [file pmed.1003537.s004.pdf]

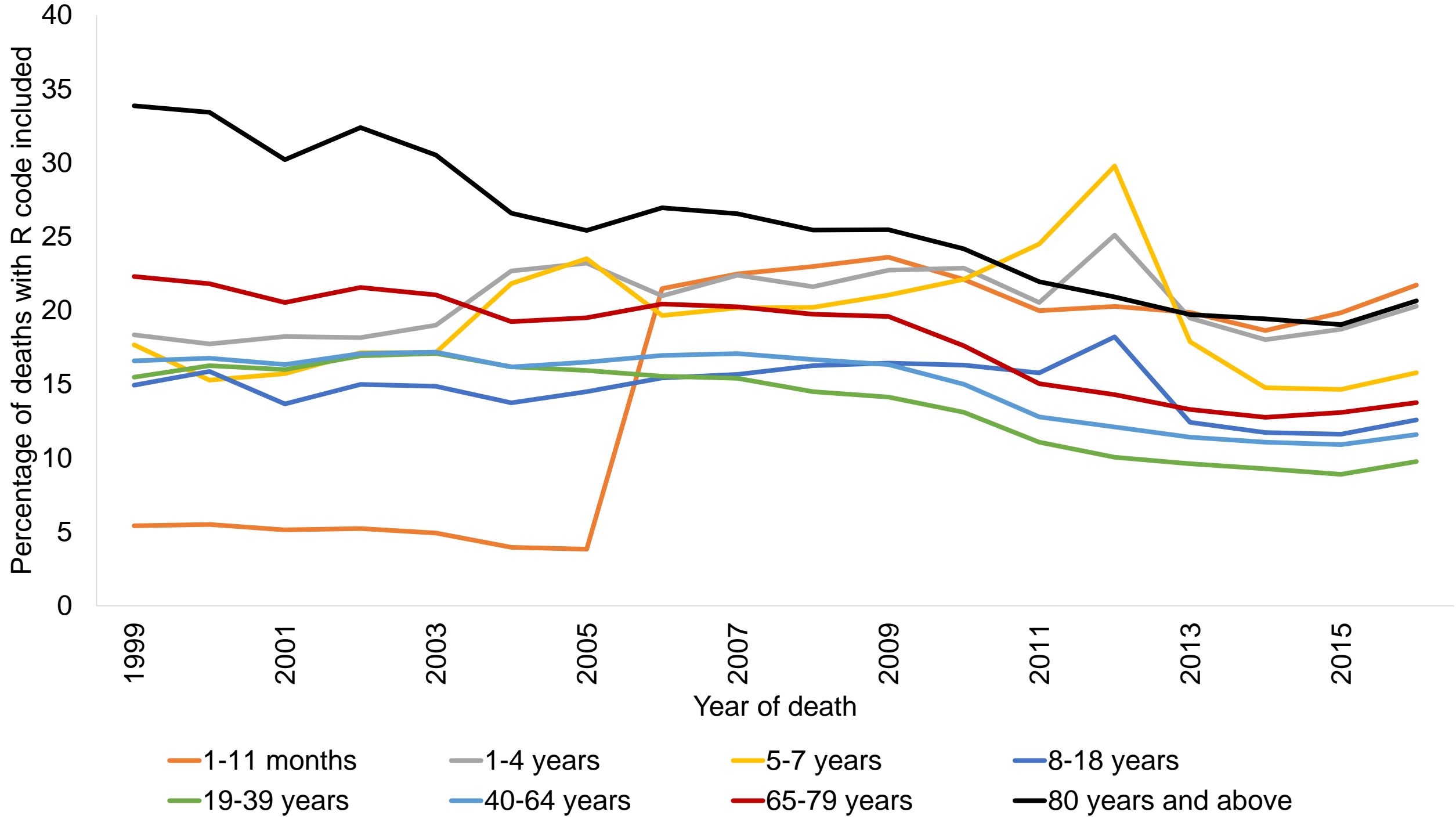

Supplement: S2 Fig — Data uncorrected for P chapter miscoding prior to 2006. (PDF) [file pmed.1003537.s005.pdf]

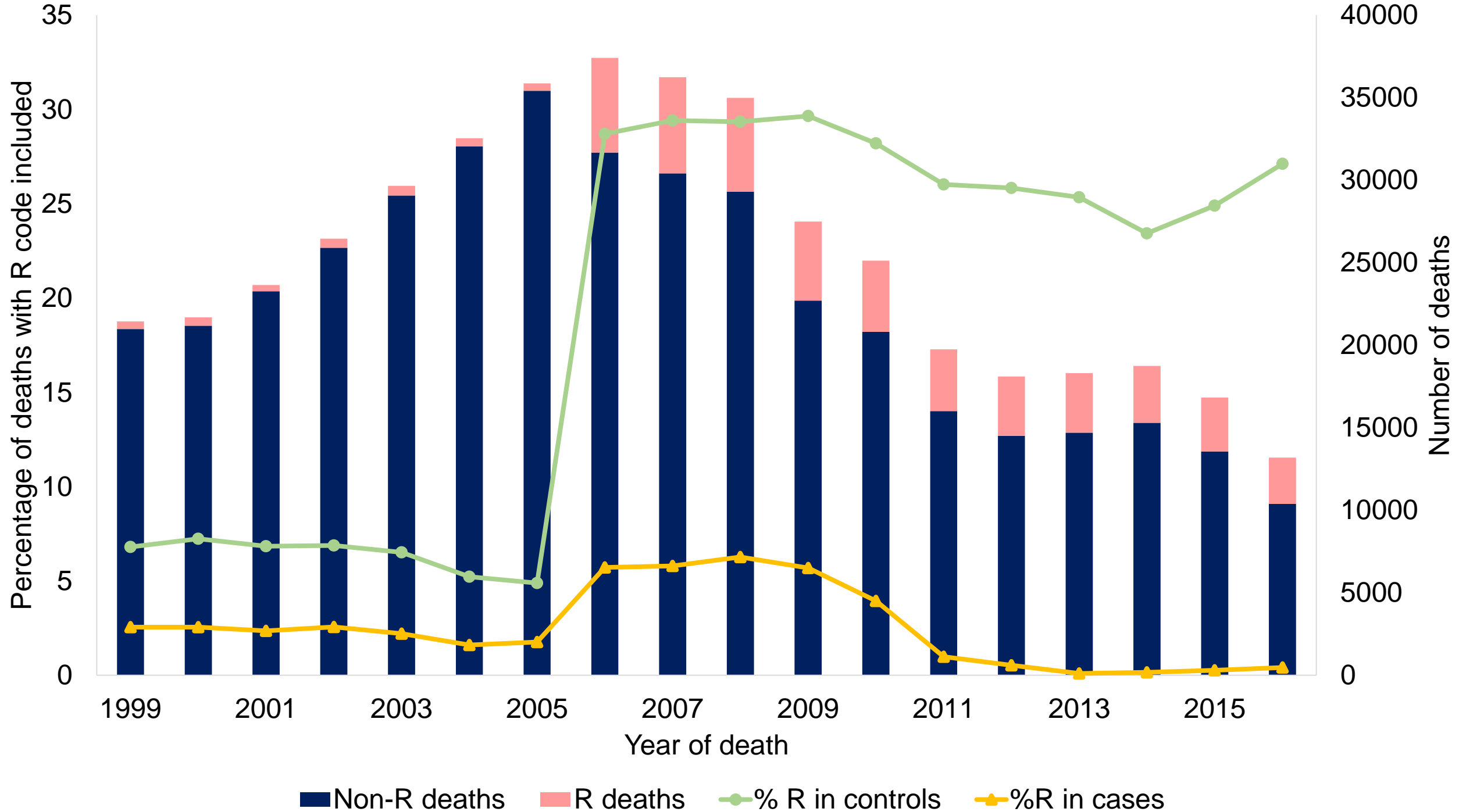

Supplement: S3 Fig — Data uncorrected for P chapter miscoding prior to 2006. (PDF) [file pmed.1003537.s006.pdf]

19-39 years main result

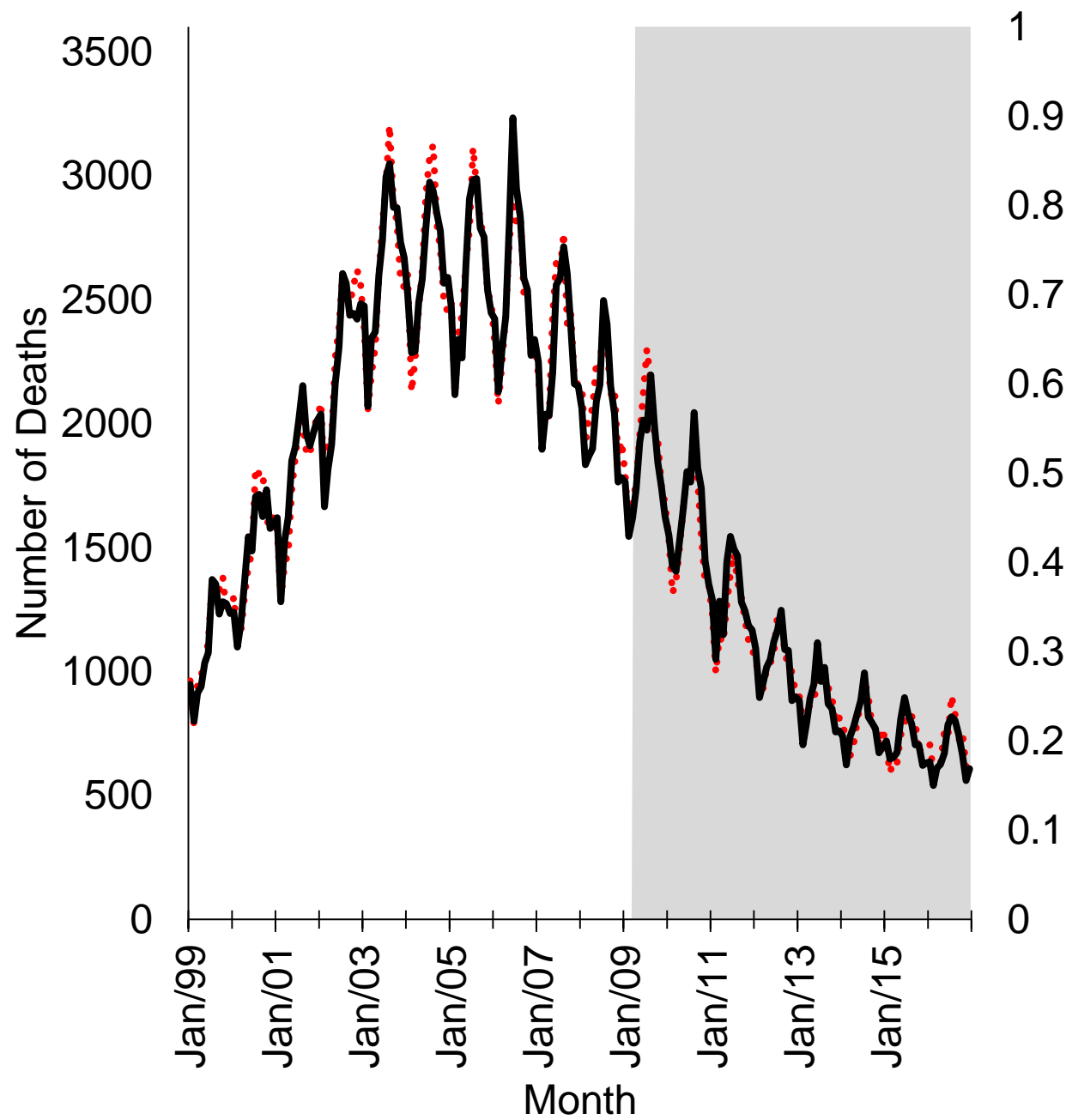

19-39 years excluding R chapter

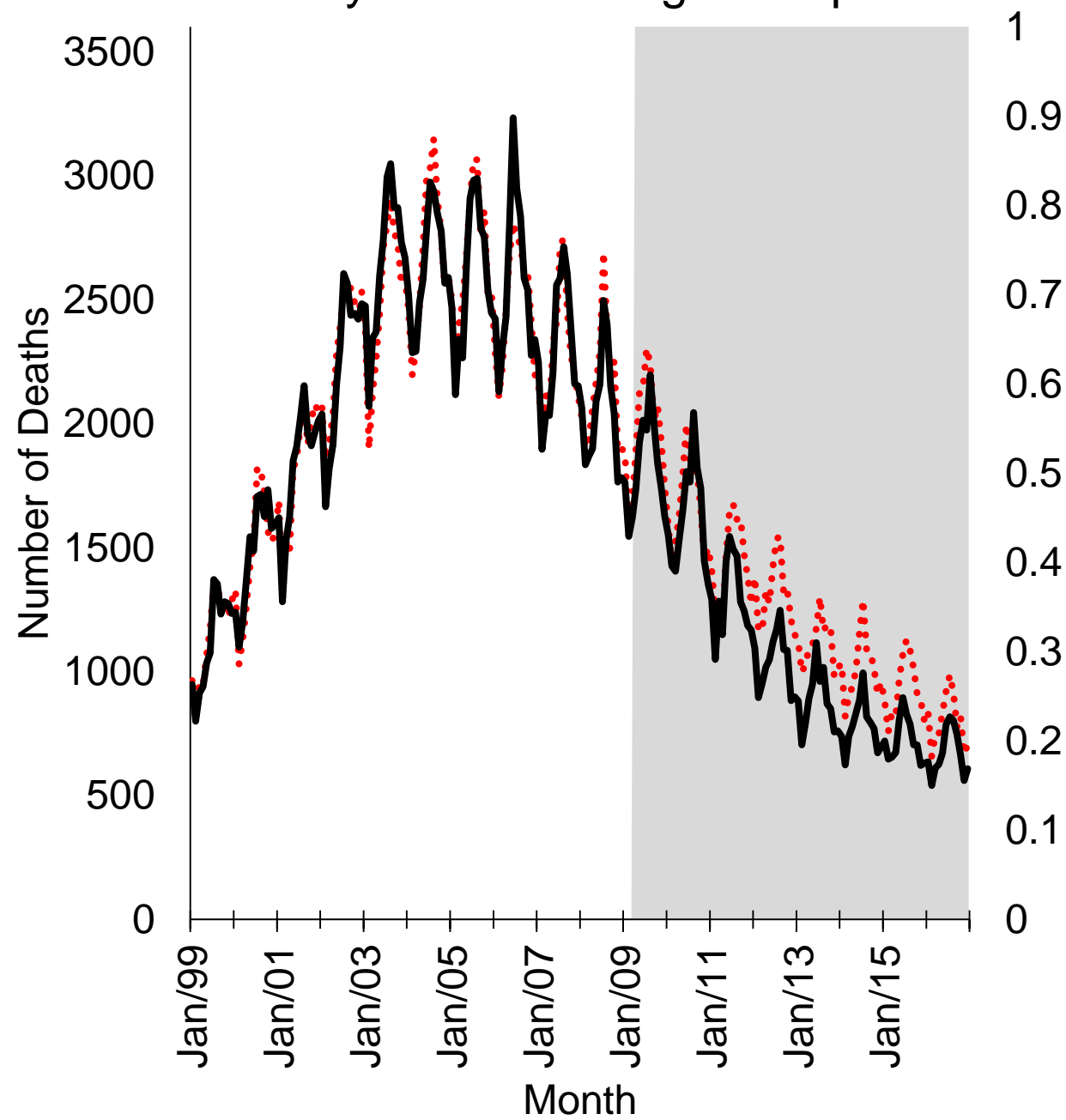

Supplement: S4 Fig — Main result (left) and the sensitivity analysis where R chapter was removed (right). Black, solid lines denote actual all-cause pneumonia. Red, dashed lines denotes counterfactual estimates. Intervention period indicated in grey. (PDF) [file pmed.1003537.s007.pdf]
