# Supplementary material for: Estimated impact of the pneumococcal conjugate vaccine on pneumonia mortality in South Africa, 1999 through 2016: An ecological modelling study
Source: PLoS Med. 2021 Feb 16;18(2):e1003537. doi: 10.1371/journal.pmed.1003537 (PMC7924778; doi:10.1371/journal.pmed.1003537)
Supplement: S1 Table — (PDF) [file pmed.1003537.s008.pdf]

S1 Table. Description of ICD codes used for outcome and composite control

**Outcome group**	**Inclusions**	**Exclusions**
All-cause pneumonia	Viral pneumonia, not elsewhere classified (J12)<br>Pneumonia due to *Streptococcus pneumoniae* (J13)<br>Pneumonia due to *Haemophilus influenzae* (J14)<br>Bacterial pneumonia, not elsewhere classified (J15)<br>Pneumonia due to other infectious organisms, not elsewhere classified (J16)<br>Pneumonia, organism unspecified (J18)<br>Congenital pneumonia (P23)	Not applicable
**Control groups**	**Inclusions**	**Exclusions**
A20_B99_a_D50_D89	Certain zoonotic bacterial diseases (A20-A28)<br>Infections with a predominantly sexual mode of transmission (A50-A64)<br>Other spirochetal diseases (A65-A69)<br>Other diseases caused by chlamydia (A70-A74)<br>Rickettsioses (A75-A79)<br>Viral infections of the central nervous system (A80-A89)<br>Arthropod-borne viral fevers and viral haemorrhagic fevers (A90-A99)<br>Viral infections characterized by skin and mucous membrane lesions (B00-B09)<br>Viral hepatitis (B15-B19)<br>Human immunodeficiency virus [HIV] disease (B20-B24)<br>Other viral diseases (B25-B34)<br>Mycoses (B35-B49)<br>Protozoal diseases (B50-B64)<br>Helminthiases (B65-B83)<br>Pediculosis, ascariasis and other infestations (B85-B89)<br>Sequelae of infectious and parasitic diseases (B90-B94)<br>Other infectious diseases (B99)<br>Infections specific to the perinatal period (P35-P39)<br>Nutritional anaemias (D50-D53)<br>Haemolytic anaemias (D55-D59)<br>Aplastic and other anaemias (D60-D64)<br>Coagulation defects, purpura and other hemorrhagic conditions (D65-D69)<br>Other diseases of blood and blood-forming organs (D70-D76)<br>Certain disorders involving the immune mechanism (D80-D89)	Streptococcal septicaemia (A40)<br>Other septicaemia (A41)<br>Bacterial infection of unspecified site (A49)<br>Bacterial sepsis of new-born (P36)
A16_A19	Tuberculosis (A16-A19)	None
A20_A48	Certain zoonotic bacterial diseases (A20-A28)<br>Other bacterial diseases (A30-A49)	
A39	Meningococcal infection (A39)	
A50_A79	Infections with a predominantly sexual mode of transmission (A50-A64)<br>Other spirochetal diseases (A65-A69)	

Control groups	Inclusions	Exclusions
	Other diseases caused by chlamydia (A70-A74)<br>Rickettsioses (A75-A79)	
A80_B34	Viral infections of the central nervous system (A80-A89)<br>Arthropod-borne viral fevers and viral hemorrhagic fevers (A90-A99)<br>Viral hepatitis (B15-B19)<br>Other viral diseases (B25-B34)	Measles (B05)<br>Rubella [German measles] (B06)<br>Human immunodeficiency virus [HIV] disease (B20-B24)
B05_B06	Measles (B05)<br>Rubella [German measles] (B06)	None
B20_B24	Human immunodeficiency virus [HIV] disease (B20-B24)	None
B35_B49	Mycoses (B35-B49)	None
B45	Cryptococcosis (B45)	None
B50_B89	Protozoal diseases (B50-B64)<br>Helminthiases (B65-B83)<br>Pediculosis, acariasis and other infestations (B85-B89)	None
B99	Other and unspecified infectious diseases (B99)	None
C00_D49	Neoplasms (C00-D48)	None
E00_E89	Endocrine, nutritional and metabolic diseases (E00-E88)	None
E10_E14	Diabetes mellitus (E10-E14)	None
E40_E46	Malnutrition (E40-E46)	None
F01_F99	Mental and behavioural disorders (F01-F99)	None
G05_G99	Diseases of the nervous system (G00-G98)	Bacterial meningitis, not elsewhere classified (G00)<br>Meningitis due to other and unspecified causes (G03)<br>Encephalitis, myelitis, and encephalomyelitis (G04)
H00_H99_excl_cj_om	Diseases of the eye and adnexa (H00-H57)	Conjunctivitis (H10)<br>Non-suppurative otitis media (H65)<br>Suppurative and unspecified otitis media (H66)<br>Mastoiditis and related conditions (H70)<br>Other disorders of middle ear and mastoid (H74)
I00_I99	Diseases of the circulatory system (I00-I99)	None
I60_I64	Subarachnoid haemorrhage (I60)<br>Intracerebral haemorrhage (I61)<br>Other non-traumatic intracranial haemorrhage (I62)	None

Control groups	Inclusions	Exclusions
	Cerebral infarction (I63)<br>Stroke, not specified as haemorrhage or infarction (I64)<br>Intracranial non-traumatic haemorrhage of foetus and new-born (P52)	
J00_J99_excl_PI_bron	Diseases of the respiratory system (J00-J98)	Influenza and pneumonia (J10-J18)<br>Other acute lower respiratory infections (J20-J22)
J20_J22	Other acute lower respiratory infections (J20-J22)	None
Inf	Influenza due to identified influenza virus (J10)	None
K00_K95	Diseases of the digestive system (K00-K92)	None
K35	Acute appendicitis (K35)	None
K80	Cholelithiasis (K80)	None
L00_L99	Diseases of the skin and subcutaneous tissue (L00-L98)	None
M00_M99	Diseases of the musculoskeletal system and connective tissue (M00-M99)	None
N00_N99	Diseases of the genitourinary system (N00-N98)	None
N39	Other disorders of urinary system (N39)	None
O00_O99	Pregnancy, childbirth and the puerperium (O00-O99)	None
P00_P15	New-born affected by maternal factors and by complications of pregnancy, labour and delivery (P00-P04)<br>Disorders related to length of gestation and foetal growth (P05-P08)<br>Birth trauma (P10-P15)	None
P05_P07	Slow foetal growth and foetal malnutrition (P05)<br>Disorders related to short gestation and low birthweight, not elsewhere classified (P07)	None
Q00_Q99	Congenital malformations, deformations and chromosomal abnormalities (Q00-Q99)	None
R00_R99	Symptoms and signs involving the circulatory and respiratory systems (R00-R09)<br>Symptoms and signs involving the digestive system and abdomen (R10-R19)<br>Symptoms and signs involving the skin and subcutaneous tissue (R20-R23)<br>Symptoms and signs involving the nervous and musculoskeletal systems (R25-R29)<br>Symptoms and signs involving the urinary system (R30-R39)<br>Symptoms and signs involving cognition, perception, emotional state and behaviour (R40-R46)<br>Symptoms and signs involving speech and voice (R47-R49)<br>Abnormal findings on examination of blood, without diagnosis (R70-R79)<br>Abnormal findings on examination of urine, without diagnosis (R80-R82)<br>Abnormal findings on examination of other body fluids, substances and tissues, without diagnosis (R83-R89)<br>Abnormal findings on diagnostic imaging and in function studies, without diagnosis (R90-R94)	None
S00_T88	Injury, poisoning and certain other consequences of external causes (S00-T98)	None
V01_Y99	External causes of morbidity and mortality (V01-Y89)	None
