# Supplementary material for: Estimated impact of the pneumococcal conjugate vaccine on pneumonia mortality in South Africa, 1999 through 2016: An ecological modelling study
Source: PLoS Med. 2021 Feb 16;18(2):e1003537. doi: 10.1371/journal.pmed.1003537 (PMC7924778; doi:10.1371/journal.pmed.1003537)
Supplement: S2 Table — Significant predictions in bold. *Observed rate in 2012–2016 compared to observed rate in 1999–2008. †Observed rate in 2012–2016 compared to predicted rate in 2012–2016, significant differences in bold. (PDF) [file pmed.1003537.s009.pdf]

S2 Table. Rate of observed and predicted (counterfactual) deaths per 100,000 population due to all-cause pneumonia in the pre-vaccine period (1999-2008) and the post-vaccine period (2012-2016)

	1999-2008		2012-2016		Changes		
	**Predicted (Range)**	**Observed (Range)**	**Predicted (Range)**	**Observed (Range)**	**Absolute Reduction* (%)**	**Modelled Reduction† (%) (95% CrI)**	**Rate ratio† (95% CrI)**
1-11 months	1 089 (830 to 1 338)	1 091 (802 to 1 314)	503 (425 to 546)	332 (256 to 362)	70	**33 (26 to 43)**	**0.67 (0.57 to 0.74)**
1-4 years	77 (55 to 91)	77 (52 to 93)	37 (29 to 43)	29 (21 to 35)	63	**23 (17 to 29)**	**0.77 (0.71 to 0.83)**
5-7 years	17 (10 to 23)	17 (9 to 23)	9 (7 to 11)	7 (4 to 12)	60	**25 (19 to 32)**	**0.75 (0.68 to 0.81)**
8-18 years	10 (8 to 12)	10 (7 to 13)	10 (9 to 11)	8 (7 to 9)	26	**23 (11 to 32)**	**0.77 (0.68 to 0.89)**
19-39 years	159 (90 to 198)	159 (89 to 201)	50 (41 to 65)	49 (39 to 65)	69	2 (-21 to 14)	0.98 (0.86 to 1.21)
40-64 years	216 (138 to 255)	215 (134 to 264)	138 (112 to 168)	140 (125 to 157)	35	-3 (-36 to 15)	1.03 (0.85 to 1.36)
65-79 years	401 (378 to 427)	401 (371 to 451)	349 (332 to 362)	371 (365 to 380)	7	-7 (-17 to 4)	1.07 (0.96 to 1.17)
≥80 years	1 286 (1 174 to 1 399)	1 283 (1 187 to 1 413)	1 242 (1 211 to 1 282)	1 327 (1 286 to 1 366)	-3	-8 (-15 to 4)	1.08 (0.96 to 1.15)

Significant predictions in bold. *Observed rate in 2012-2016 compared to observed rate in 1999-2008. †Observed rate in 2012-2016 compared to predicted rate in 2012-2016, significant differences in bold.
