# Supplementary material for: Estimated impact of the pneumococcal conjugate vaccine on pneumonia mortality in South Africa, 1999 through 2016: An ecological modelling study
Source: PLoS Med. 2021 Feb 16;18(2):e1003537. doi: 10.1371/journal.pmed.1003537 (PMC7924778; doi:10.1371/journal.pmed.1003537)
Supplement: S3 Table — (PDF) [file pmed.1003537.s010.pdf]

S3 Table. Top three control groups included in main synthetic control analysis by age

	Greatest inclusion control (inclusion probability)	Second greatest inclusion control (inclusion probability)	Third greatest inclusion control (inclusion probability)
1-11 months	B50_B89 (0.990)	B50_B89 (0.990)	R00_R99 (0.845)
1-4 years	A16_A19 (1.000)	A16_A19 (1.000)	E00_E89 (0.852)
5-7 years	A16_A19 (1.000)	A16_A19 (1.000)	A20_B99_a_D50_D89 (0.866)
8-18 years	A16_A19 (0.991)	A16_A19 (0.991)	V01_Y99 (0.306)
19-39 years	R00_R99 (1.000)	R00_R99 (1.000)	O00_O99 (0.927)
40-64 years	A16_A19 (0.999)	A16_A19 (0.999)	B20_B24 (0.573)
65-79 years	J00_J99_excl_PI_bron (0.992)	J00_J99_excl_PI_bron (0.992)	I60_I64 (0.911)
≥80 years	R00_R99 (0.951)	R00_R99 (0.951)	J20_J22 (0.926)
