# Supplementary material for: Estimated impact of the pneumococcal conjugate vaccine on pneumonia mortality in South Africa, 1999 through 2016: An ecological modelling study
Source: PLoS Med. 2021 Feb 16;18(2):e1003537. doi: 10.1371/journal.pmed.1003537 (PMC7924778; doi:10.1371/journal.pmed.1003537)
Supplement: S4 Table — Rate ratio (RR), 95% credible interval (CrI) in brackets, significant predictions in bold. (PDF) [file pmed.1003537.s011.pdf]

S4 Table. Sensitivity analysis of changes in deaths for all-cause pneumonia mortality (rate ratio) by removing the controls with highest inclusion probabilities, in the post-vaccine period (2012-2016), South Africa

	RR using all control diseases	Control 1	RR if control 1 excluded	Control 2	RR if control 1 and 2 excluded	Control 3	RR if control 1, 2, and 3 excluded
1-11 months	**0.67 (0.57 to 0.74)**	B50_B89	**0.65 (0.60 to 0.69)**	R00_R99	**0.56 (0.49 to 0.60)**	J00_J99_excl_PI_bron	**0.65 (0.58 to 0.71)**
1-4 years	**0.77 (0.71 to 0.83)**	A16_A19	**0.56 (0.53 to 0.60)**	J00_J99_excl_PI_bron	**0.60 (0.52 to 0.66)**	J20_J22	**0.55 (0.52 to 0.62)**
5-7 years	**0.75 (0.68 to 0.81)**	A16_A19	**0.69 (0.60 to 0.77)**	A20_B99_a_D50_D89	**0.58 (0.54 to 0.66)**	A80_B34	**0.57 (0.50 to 0.63)**
8-18 years	**0.77 (0.68 to 0.89)**	A16_A19	**0.62 (0.58 to 0.71)**	A20_B99_a_D50_D89	**0.69 (0.62 to 0.75)**	J00_J99_excl_PI_bron	**0.65 (0.59 to 0.69)**
19-39 years	0.98 (0.86 to 1.21)	R00_R99	0.99 (0.96 to 1.03)	A16_A19	**0.68 (0.58 to 0.97)**	J00_J99_excl_PI_bron	**0.60 (0.54 to 0.64)**
40-64 years	1.03 (0.85 to 1.36)	A16_A19	0.98 (0.94 to 1.02)	J00_J99_excl_PI_bron	0.98 (0.94 to 1.03)	R00_R99	**0.67 (0.64 to 0.93)**
65-79 years	1.07 (0.96 to 1.17)	J00_J99_excl_PI_bron	0.99 (0.95 to 1.03)	I00_I99	1.01 (0.97 to 1.40)	Inf	1.44 (1.02 to 1.60)
≥80 years	1.08 (0.96 to 1.15)	R00_R99	0.98 (0.95 to 1.01)	J00_J99_excl_PI_bron	0.97 (0.83 to 1.01)	I00_I99	1.03 (0.98 to 1.07)

Rate ratio (RR), 95% credible interval (CrI) in brackets, significant predictions in bold
