# Supplementary material for: Estimated impact of the pneumococcal conjugate vaccine on pneumonia mortality in South Africa, 1999 through 2016: An ecological modelling study
Source: PLoS Med. 2021 Feb 16;18(2):e1003537. doi: 10.1371/journal.pmed.1003537 (PMC7924778; doi:10.1371/journal.pmed.1003537)
Supplement: S5 Table — Rate ratio (RR), 95% credible interval (CrI) in brackets, significant predictions in bold. (PDF) [file pmed.1003537.s012.pdf]

S5 Table. Sensitivity analysis of changes in deaths for all-cause pneumonia mortality (rate ratio) by removing certain controls, in the post-vaccine period (2012-2016), South Africa

	RR Main model	RR Excluding J20_J22	RR Excluding J ICD chapter, but including J20_J22	RR Excluding entire J ICD chapter	RR Excluding entire R ICD chapter
1-11 months	**0.67 (0.57 to 0.74)**	**0.67 (0.56 to 0.74)**	**0.68 (0.61 to 0.75)**	**0.68 (0.61 to 0.74)**	**0.66 (0.55 to 0.73)**
1-4 years	**0.77 (0.71 to 0.83)**	**0.77 (0.71 to 0.83)**	**0.77 (0.71 to 0.83)**	**0.77 (0.71 to 0.83)**	**0.77 (0.70 to 0.83)**
5-7 years	**0.75 (0.68 to 0.81)**	**0.75 (0.68 to 0.81)**	**0.74 (0.67 to 0.81)**	**0.74 (0.67 to 0.81)**	**0.75 (0.68 to 0.81)**
8-18 years	**0.77 (0.68 to 0.89)**	**0.77 (0.68 to 0.89)**	**0.77 (0.68 to 0.89)**	**0.77 (0.68 to 0.89)**	**0.77 (0.68 to 0.89)**
19-39 years	0.98 (0.86 to 1.21)	0.98 (0.86 to 1.20)	0.98 (0.85 to 1.21)	0.98 (0.86 to 1.19)	**0.81 (0.68 to 0.97)**
40-64 years	1.03 (0.85 to 1.36)	1.03 (0.85 to 1.37)	1.04 (0.85 to 1.40)	1.04 (0.85 to 1.40)	1.02 (0.85 to 1.17)
65-79 years	1.07 (0.96 to 1.17)	1.07 (0.96 to 1.17)	0.97 (0.93 to 1.14)	0.97 (0.92 to 1.13)	1.07 (0.96 to 1.10)
≥80 years	1.08 (0.96 to 1.15)	0.97 (0.90 to 1.10)	1.09 (0.96 to 1.16)	0.98 (0.88 to 1.11)	0.96 (0.89 to 1.03)

Rate ratio (RR), 95% credible interval (CrI) in brackets, significant predictions in bold
