# Supplementary material for: Estimated impact of the pneumococcal conjugate vaccine on pneumonia mortality in South Africa, 1999 through 2016: An ecological modelling study
Source: PLoS Med. 2021 Feb 16;18(2):e1003537. doi: 10.1371/journal.pmed.1003537 (PMC7924778; doi:10.1371/journal.pmed.1003537)
Supplement: S6 Table — Rate ratio (RR), 95% credible interval (CrI) in brackets, significant predictions in bold. (PDF) [file pmed.1003537.s013.pdf]

S6 Table. Sensitivity analysis of changes in deaths for all-cause pneumonia mortality (rate ratio) on unadjusted data (no reclassification of P (neonatal) codes in children aged 1-11 months, and not grouping D50-D89 with A10-B99), in the post-vaccine period (2012-2016), South Africa

	Main results		No reclassification of P codes to other groups		No reclassification of P codes, excluding D50-D89 in A20-B99	
	**RR (95% CrI)**	**Top 3 controls**	**RR (95% CrI)**	**Top 3 controls**	**RR (95% CrI)**	**Top 3 controls**
1-11 months	**0.67 (0.57, 0.74)**	B50_B89, R00_R99, E40_E46	**0.59 (0.54, 0.63)**	A16_A19, A20_B99_a_D50_D89, B50_B89	**0.67 (0.55, 0.77)**	B50_B89, A16_A19, D50_D89
1-4 years	**0.77 (0.71, 0.83)**	A16_A19, E00_E89, B50_B89	**0.77 (0.7, 0.83)**	A16_A19, E00_E89, B50_B89	**0.78 (0.7, 0.87)**	A16_A19, E00_E89, B50_B89
5-7 years	**0.75 (0.68, 0.81)**	A16_A19, A20_B99_a_D50_D89, A80_B34	**0.75 (0.67, 0.81)**	A16_A19, A20_B99_a_D50_D89, A80_B34	**0.73 (0.66, 0.8)**	A16_A19, A20_B99_excl_bac, A80_B34
8-18 years	**0.77 (0.68, 0.89)**	A16_A19, V01_Y99, A20_B99_a_D50_D89	**0.77 (0.68, 0.89)**	A16_A19, V01_Y99, A20_B99_a_D50_D89	**0.77 (0.71, 0.89)**	A16_A19, V01_Y99, E10_E14
19-39 years	0.98 (0.86, 1.21)	R00_R99, O00_O99, A16_A19	0.98 (0.86, 1.22)	R00_R99, A16_A19, O00_O99	0.99 (0.86, 1.23)	R00_R99, A16_A19, O00_O99
40-64 years	1.03 (0.85, 1.36)	A16_A19, B20_B24, J00_J99_excl_PI_bron	1.03 (0.85, 1.37)	A16_A19, B20_B24, J00_J99_excl_PI_bron	1.03 (0.85, 1.36)	A16_A19, B20_B24, J00_J99_excl_PI_bron
65-79 years	1.07 (0.96, 1.17)	J00_J99_excl_PI_bron, I60_I64, I00_I99	1.07 (0.96, 1.17)	J00_J99_excl_PI_bron, I60_I64, I00_I99	1.07 (0.96, 1.16)	J00_J99_excl_PI_bron, I60_I64, I00_I99
≥80 years	1.08 (0.96, 1.15)	R00_R99, J20_J22, J00_J99_excl_PI_bron	1.08 (0.95, 1.14)	R00_R99, J20_J22, J00_J99_excl_PI_bron	1.08 (0.95, 1.14)	R00_R99, J20_J22, J00_J99_excl_PI_bron

Rate ratio (RR), 95% credible interval (CrI) in brackets, significant predictions in bold
