# Supplementary material for: Estimated impact of the pneumococcal conjugate vaccine on pneumonia mortality in South Africa, 1999 through 2016: An ecological modelling study
Source: PLoS Med. 2021 Feb 16;18(2):e1003537. doi: 10.1371/journal.pmed.1003537 (PMC7924778; doi:10.1371/journal.pmed.1003537)
Supplement: S7 Table — Rate ratio (RR), 95% credible interval (CrI) in brackets, significant predictions in bold. (PDF) [file pmed.1003537.s014.pdf]

S7 Table. Sensitivity analysis of changes in deaths for all-cause pneumonia mortality (rate ratio) on 2006-2016 analysis, by removing the controls with highest inclusion probabilities, in the post-vaccine period (2012-2016), South Africa

	RR using all control diseases	Control 1	RR if control 1 excluded	Control 2	RR if control 1 and 2 excluded	Control 3	RR if control 1, 2, and 3 excluded
1-11 months	1.13 (0.51 to 1.57)	R00_R99	0.98 (0.46 to 1.03)	J00_J99_excl_PI_bron	1.01 (0.97 to 1.05)	J20_J22	0.95 (0.59 to 1.02)
1-4 years	**0.69 (0.59 to 0.90)**	E00_E89	**0.65 (0.57 to 0.89)**	J00_J99_excl_PI_bron	0.71 (0.48 to 1.05)	R00_R99	0.72 (0.47 to 1.05)
5-7 years	**0.74 (0.60 to 0.95)**	A20_B99_a_D50_D89	1.00 (0.74 to 1.08)	R00_R99	0.71 (0.43 to 1.11)	A16_A19	**0.47 (0.41 to 0.57)**
8-18 years	**0.69 (0.59 to 0.89)**	B50_B89	0.96 (0.66 to 1.02)	A16_A19	0.96 (0.63 to 1.01)	R00_R99	**0.65 (0.61 to 0.71)**
19-39 years	0.84 (0.62 to 1.24)	R00_R99	0.98 (0.95 to 1.01)	J00_J99_excl_PI_bron	0.97 (0.93 to 1.02)	A16_A19	0.53 (0.44 to 0.89)
40-64 years	**0.74 (0.64 to 0.90)**	J00_J99_excl_PI_bron	1.00 (0.66 to 1.02)	I00_I99	1.01 (0.96 to 2.21)	R00_R99	1.00 (0.96 to 1.48)
65-79 years	1.02 (0.90 to 1.12)	J00_J99_excl_PI_bron	0.98 (0.95 to 1.02)	I00_I99	1.00 (0.96 to 1.77)	I60_I64	0.99 (0.95 to 1.84)
≥80 years	1.34 (0.86 to 1.46)	R00_R99	0.99 (0.88 to 1.03)	J00_J99_excl_PI_bron	0.98 (0.83 to 1.04)	I00_I99	1.01 (0.94 to 1.05)

Rate ratio (RR), 95% credible interval (CrI) in brackets, significant predictions in bold
