# Supplementary material for: Estimated impact of the pneumococcal conjugate vaccine on pneumonia mortality in South Africa, 1999 through 2016: An ecological modelling study
Source: PLoS Med. 2021 Feb 16;18(2):e1003537. doi: 10.1371/journal.pmed.1003537 (PMC7924778; doi:10.1371/journal.pmed.1003537)
Supplement: S8 Table — Rate ratio (RR), 95% credible interval (CrI) in brackets, significant predictions in bold. (PDF) [file pmed.1003537.s015.pdf]

**S8 Table. Sensitivity analysis of changes in deaths for all-cause pneumonia mortality (rate ratio) by removing certain years, or aggregating by trimester, in the post-vaccine period (2012-2016), South Africa**

| | RR Main model | RR Start 2006 | RR Excl 2009 | RR Excl 2015-2016 | RR Trimester aggregated |
|---|---|---|---|---|---|
| 1-11 months | **0.67 (0.57 to 0.74)** | 1.13 (0.51 to 1.57) | **0.63 (0.50 to 0.71)** | **0.69 (0.59 to 0.76)** | **0.69 (0.63 to 0.75)** |
| 1-4 years | **0.77 (0.71 to 0.83)** | **0.69 (0.59 to 0.90)** | **0.76 (0.68 to 0.82)** | **0.78 (0.72 to 0.84)** | **0.72 (0.66 to 0.79)** |
| 5-7 years | **0.75 (0.68 to 0.81)** | **0.74 (0.60 to 0.95)** | **0.71 (0.65 to 0.79)** | **0.80 (0.72 to 0.88)** | **0.74 (0.67 to 0.81)** |
| 8-18 years | **0.77 (0.68 to 0.89)** | **0.69 (0.59 to 0.89)** | **0.76 (0.66 to 0.89)** | **0.76 (0.67 to 0.88)** | **0.76 (0.67 to 0.80)** |
| 19-39 years | 0.98 (0.86 to 1.21) | 0.84 (0.62 to 1.24) | 0.98 (0.85 to 1.23) | 0.99 (0.86 to 1.23) | 0.96 (0.84 to 1.25) |
| 40-64 years | 1.03 (0.85 to 1.36) | **0.74 (0.64 to 0.90)** | 0.89 (0.84 to 1.25) | 0.98 (0.83 to 1.30) | 1.03 (0.85 to 1.22) |
| 65-79 years | 1.07 (0.96 to 1.17) | 1.02 (0.90 to 1.12) | 1.07 (0.97 to 1.13) | 1.04 (0.96 to 1.14) | 0.99 (0.94 to 1.09) |
| ≥80 years | 1.08 (0.96 to 1.15) | 1.34 (0.86 to 1.46) | 1.08 (0.95 to 1.15) | 1.05 (0.95 to 1.12) | 1.06 (0.92 to 1.13) |

Rate ratio (RR), 95% credible interval (CrI) in brackets, significant predictions in bold
