# Supplementary material for: Estimated impact of the pneumococcal conjugate vaccine on pneumonia mortality in South Africa, 1999 through 2016: An ecological modelling study
Source: PLoS Med. 2021 Feb 16;18(2):e1003537. doi: 10.1371/journal.pmed.1003537 (PMC7924778; doi:10.1371/journal.pmed.1003537)
Supplement: S9 Table — Rate ratio (RR), 95% credible interval (CrI) in brackets, significant predictions in bold. (PDF) [file pmed.1003537.s016.pdf]

S9 Table. Changes in deaths for all-cause pneumonia mortality (rate ratio) from synthetic control (SC) and interrupted time series (ITS) analysis, in the post-vaccine period (2012-2016), South Africa

	ITS	SC
1-11 months	**0.66 (0.59, 0.74)**	**0.67 (0.57 to 0.74)**
1-4 years	**0.29 (0.26, 0.33)**	**0.77 (0.71 to 0.83)**
5-7 years	**0.31 (0.28, 0.36)**	**0.75 (0.68 to 0.81)**
8-18 years	**0.22 (0.18, 0.26)**	**0.77 (0.68 to 0.89)**
19-39 years	**0.47 (0.42, 0.51)**	0.98 (0.86 to 1.21)
40-64 years	**0.23 (0.20, 0.27)**	1.03 (0.85 to 1.36)
65-79 years	**0.39 (0.35, 0.44)**	1.07 (0.96 to 1.17)
≥80 years	1.00 (0.90, 1.10)	1.08 (0.96 to 1.15)

Rate ratio (RR), 95% credible interval (CrI) in brackets, significant predictions in bold
