# Supplementary material for: Estimated impact of the pneumococcal conjugate vaccine on pneumonia mortality in South Africa, 1999 through 2016: An ecological modelling study
Source: PLoS Med. 2021 Feb 16;18(2):e1003537. doi: 10.1371/journal.pmed.1003537 (PMC7924778; doi:10.1371/journal.pmed.1003537)
Supplement: Attachment — Submitted filename: Responses Kleynhans PCV impact mortality RSA Subm 2.docx [file pmed.1003537.s017.docx]

13 November 2020

Please find herewith the submission of the revised manuscript, and below detailed responses to the reviewer’s comments. Original comments shown in black font and responses in green.

**Requests from the editors:**

**Academic editor comments**

"I would recommend major revision, with more detail on methods and the exact way they constructed the synthetic cohort would help. Reviewer 2 has asked for minor revisions but her comments are tougher than the others. I also think they need to explain the potential impact of the PCV 13 vs 10 transition as well as the potential increase of non-vaccine serotypes in SA".

Thank you for these comments. We have included more detail on methods and discussion of the potential impact of the PCV 13 vs 10 transition as well as the potential increase of non-vaccine serotypes in SA. Details of these changes are included as part of detailed responses to reviewers outlined below.

**Internal editorial requests (formatting/writeup)**

1. We'd recommend revising the title per PLOS Medicine's usual style, normally this includes some indication of the study design/methodological approach in the subtitle of the title (usually after a colon). In this case would "ecological analysis" or "modelling study" be appropriate (the authors clearly are best placed to decide here on what is most indicative).

Title changed to: “Impact of the pneumococcal conjugate vaccine on pneumonia mortality in South Africa, 1999 through 2016**: an ecological modelling study**”

2. At this stage, we ask that you include a short, non-technical Author Summary of your research to make findings accessible to a wide audience that includes both scientists and non-scientists. The Author Summary should immediately follow the Abstract in your revised manuscript. This text is subject to editorial change and should be distinct from the scientific abstract. Please see our author guidelines for more information: <https://journals.plos.org/plosmedicine/s/revising-your-manuscript#loc-author-summary>

The author summary was added.

3. If possible please reformat the citation style into PLOS Medicine's format (should be straight forward if using referencing software) - this should use callouts formatted as sequential numerals in square brackets (not superscript). Many thanks

Reference styled changed to PLoS.

4. Minor typo in the discussion (trail not trial) -
   a. "To our knowledge, this is the first study on the impact of PCV on pneumonia mortality in all age groups on a national scale in Africa. A 16% reduction in all-cause childhood mortality was observed during a randomized control trail evaluating PCV9 in Upper and Central River Division of The Gambia.32"

Trail updated to Trial, line 360, page 12. Please note all line numbers from here on refer to document with tracked changes.

5. We'd ask the authors to clarify in the revised paper whether the analytical approach followed in this study corresponded to one laid out in a prospective protocol or analysis plan. Please state this (either way) early in the Methods section.
   a. If a prospective analysis plan (from your funding proposal, IRB or other ethics committee submission, study protocol, or other planning document written before analyzing the data) was used in designing the study, please include the relevant prospectively written document with your revised manuscript as a Supporting Information file to be published alongside your study, and cite it in the Methods section. A legend for this file should be included at the end of your manuscript.

Legend for supporting information added at the end of the manuscript, line 429 page 20.

b. If no such document exists, please make sure that the Methods section transparently describes when analyses were planned, and when/why any data-driven changes to analyses took place.
c. In either case, changes in the analysis-- including those made in response to peer review comments-- should be identified as such in the Methods section of the paper, with rationale.

The following was added to the first paragraph of the *Estimating the impact of PCV on all-cause pneumonia mortality* methods section (line 130 page 8): “**As laid out in the initial analysis plan (see supplement)**, we utilized a synthetic control approach to adjust for disease trends unrelated to the vaccine as described by Bruhn, et al. [10] **The only change to the analysis plan was to include 2016, as the data became available prior to the start of analysis.**” Furthermore, this analysis plan was included in the supplementary information, page 20.

-----------------------------------------------------------

**Comments from the reviewers:**

**Reviewer #1: Statistical review**

This paper reports an analysis investigating to what extent the introduction of pneumococcal conjugate vaccine reduced the mortality due to pneumonia in South Africa. An interesting analysis approach is used, based on using a synthetic control group from other mortality reasons. My most major comment (see comment 3) is that I think more information is required on the method.

I have some comments, listed below:

1. Abstract: it would be useful to add a brief description of the analysis methods (i.e that it is Bayesian).

We’ve added to the Methods and findings section of the abstract (line 38 page 3): “We created a composite (synthetic) control using **Bayesian variable selection with** non-diarrheal, non-pneumonia and non-pneumococcal deaths to estimate the number of expected all-cause pneumonia deaths in the absence of PCV introduction post 2009.”

2. Page 4 - it may be common language in vaccine settings but I didn't follow what 'no catch up' and 'limited catch up' meant. Is this referring to not giving extra doses to children who were slightly too old at the introduction of the vaccination programme?

To assist with clarifying, we’ve included the following in paragraph 2 of the introduction, line 92 – 96 page 6: “The seven-valent pneumococcal conjugate vaccine (PCV7) was introduced in the South African Expanded Programme on Immunization in 2009 with no catch-up **doses** **offered to children older than 6 weeks at the time of vaccine introduction (only children aged 6 weeks or younger on 1 April 2009 were eligible for PCV7).** The country transitioned to the 13-valent (PCV13) vaccine in 2011 with a limited catch-up **where children who had received two or more doses of PCV7 received a dose of PCV13 at 18 months and children aged >18 to 36 months received a single dose of PCV13**.”

3. Statistical methods: I think the authors give intuition over the approach they have used, and provide full code for reproducing the results, which is commendable. I think some of the steps of the process could do with fleshing out a bit in the paper (or supplementary material). I think the text in the second paragraph of page 7 (starting 'We fitted a regression…') requires some more details:

We added more details to the methods in the main manuscript as requested and described below, but also included further details in the supporting information.

a) 'time series of all possible controls as covariates' - I did not follow how many covariates this would be or how they would be produced. Is this part of the model building, with each possible cause of death a potential control?

In response, we added in line 155, page 9: “~~Control~~ **All** causes of deaths **classified as controls** were grouped based on ICD-10 chapter (Table S1) **based on the ICD-10 code listed in any cause of death variable** and aggregated to the monthly time scale” And line 169 page 9: “**The synthetic control model used the Bayesian variable selection (with spike-and-slab priors) to determine the contribution to 38 control diseases (all log-transformed, Table S1 in supporting information).**”

b) I think more details over the Bayesian variable selection model should be provided - is this some sort of Bayesian sparse regression model?

We added line 177 page 9: “We **then** fitted a regression model to the monthly pre-vaccine number of pneumonia deaths (log-transformed) **using a Bayesian structural time-series model.**”

c) Does the BVS model result in uncertainty? It's implied that a deterministic equation is used for the expected number of deaths. Or is this done deterministically within each MCMC sample with the uncertainty dealt with that way?

To clarify we added to paragraph 2 page: “We then fitted a regression model to the monthly pre-vaccine number of pneumonia deaths (log-transformed) using a Bayesian structural time-series model. **This allowed us to quantify uncertainty associated with variable selection and variation in the data.** This process generated an equation that described the relationship between the control variables and pneumonia deaths in the absence of the vaccine.[10] A composite control was generated by plugging in the observed values of the control diseases during the post-PCV period into this equation (equation S1). The output from this model gives provided an estimate for the expected number of pneumonia deaths if the intervention (PCV) had not been introduced for the post-PCV period (the counterfactual). … **We drew 10 000 Markov Chain Monte Carlo (MCMC) samples, which allowed us to quantify uncertainty associated with variable selection and variation in the data.”**

d) Is the relationship between the selected control conditions and pneumonia stable over time in the pre-vaccination period? Would this have an impact on the results and level of uncertainty in the post-vaccination period?

The synthetic control approach is based on the assumption that the relationship between the control condition and outcome condition is stable in the pre-vaccine period. Although this assumption was mentioned in the ‘Exclusions from controls’ section in the supplementary material, all assumptions are now detailed in the new addition to the supplement ‘Detailed Methods’.

4. Results: The authors present plenty of sensitivity analyses. One set of analyses excluded a couple of different years. I didn't really follow the rationale for picking those years. Given that pneumonia mortality increased until 2006 and then decreased, I would have been interested to see whether excluding all years before 2006 led to any changes to the results.

We’ve included this sensitivity analysis as suggested and added to the results, line 277 page 14: “**When we excluded 1999-2005, results were similar for all age groups, except 1-11 months where no effect was seen and those 40-64 years where a 26% (10% - 36%) reduction in mortality was observed (Table S5).”,** and the discussion line 338 page 16: **“When we reduced the pre-vaccine period for the analysis, using data from 2006 onwards to exclude the period in which pneumonia mortality was increasing – most likely attributable to South Africa’s HIV epidemic,[31] we observed no effect of PCV in those aged 1-11 months and 26% reduction in the 40-64 year olds, results unique to this specific sensitivity analysis with no similar signals in any of the other sensitivity analyses. It is interesting to note that the sudden change in R coding practices for the 1-11 months-old occurred in 2006, but we do not believe this to be the reason for this difference as excluding the R chapter from this same analysis still did not result in a measured effect. In the backdrop of upscaling prevention of mother to child HIV transmission, and by not considering prior years, the added effect of PCV may have been too small to measure using an ecological study. The effect observed in the 40-46-year-old group when excluding pre-2006 data could possibly be due to an artefact of the control choice.”**

James Wason

-----------------------------------------------------------

**Reviewer #2:**

In this paper the authors analyze the effectiveness of PCV 10 on pneumonia and mortality among all age groups in South Africa. The paper is clearly written and the data presented are sound. I have only a few remarks:

1. I could not follow the link to "Oliveira LHd, Shioda K, Valenzuela MT, et al. Declines in Pneumonia Mortality Following the Introduction of Pneumococcal Conjugate Vaccines in Latin American and Caribbean Countries. (05/31/2019 20:53:28) Available at SSRN: https://ssrncom/abstract=3398536 2019." Please update and check all other links to see if they are still valid

Reference updated

2. Apart from rotavirus vaccines, were there any other vaccines introduced in the country during the study period, considering all age groups? What could be there potential effect?

No other changes were made to the South African vaccine schedules that would have had an effect on the results. We’ve added the following to the Exclusion form controls section in the supplementary material: “**Other changes in the South Africa expanded programme on immunisation during the study period was the introduction of Haemophilus influenzae type b (Hib) vaccine in 1999, which would have also led to a reduction in pneumonia deaths prior to 2009 of which the synthetic control approach would have controlled for. The only other changes were swopping whole-cell pertussis vaccine to acellular vaccine in 2009, and opting for the hexavalent vaccine (polio, diphtheria, tetanus, pertussis, Hib) in 2015 by replacing the 5-in-1 vaccine and Hepatitis B vaccines.[3] We did not expect these changes to have an influence on our analysis**”

3. When explaining the selection of the age-strata used in the study, we are given the impression that 5-7-year-old kids could only have been vaccinated in catch up campaigns. However, as the years accumulate after vaccination start, most of the vaccinated ones in this age group must have received their vaccination as scheduled by the NIP in previous years of their life. Please comment the vaccine cohort effect and its implication for the interpretation of results.

Age strata were chosen based on eligibility for vaccination at the final year of study, and the 5-7-year-old group was a mix of those who could have received the vaccine in catch-up campaigns or through routine vaccination. For clarity this was added to the methods in the supplement: “Age strata in children were selected to assess the impact of PCV, based on ~~age~~ **year of birth**, on those eligible for routine vaccination (1 month to 4 years), those who could possibly have been vaccinated in catch-up campaigns (**born before February 2009**) or routine vaccine (**born February 2009 – 2011**, 5-7 years) and those not eligible for vaccination (born before 2009, 8-18 years) **in 2016, the final year of data included in the analysis**.” We also recognised this as a limitation, line 399 page 18: “**We were also not able to differentiate between direct and indirect vaccine effects in the 5-7-year-old age group as this grouped contained a mix of individuals who by 2016 might not have been vaccinated, or could have been vaccinated as part of a catch-up campaign or routine vaccination schedule.**”

4. "Control causes of deaths were grouped based on ICD-10 chapter". In order to do this grouping, did you use the "underlying cause of death"? Clarify.

To clarify we added in line 155 page 9: “~~Control~~ **All** causes of deaths **classified as controls** were grouped based on ICD-10 chapter (Table S1) **based on the ICD-10 code listed in any cause of death variable** and aggregated to the monthly time scale. **A single death could be counted in two or more groups, for example if C61 was listed as cause A and N39 as cause B, the death counted towards both the C00_D49 and N39 groups.**”

5. The following paragraph is not clear and has no reference: "We weighted the controls using Bayesian variable selection based on their ability to jointly fit the trend of the outcome variable (pneumonia deaths) in the pre-vaccine period. We drew 10 000 samples using Markov Chain Monte Carlo (MCMC). This process generates an equation that describes the relationship between the control variables and pneumonia deaths in the absence of the vaccine." If the reference to this sentence is that of Bruhn et al mentioned several paragraphs before, it should be referenced again. The methodology, even as described in this paper, is very complex and should be more easily explained to the broad readership of this journal. How were the controls weighted? Did you also control for prior trends and seasonality?

Bruhn reference added. Technicality of methods in the main manuscript was reduced and additional information on methods added to the supporting information.

6. In fact, many vaccine impact studies have been done without the synthetic control approach. Are all these studies biased and wrong? Apart from the sensitivity analysis which you have already performed, would it be of interest to see the impact measurements obtained with and without the use of this methodology to see how different they are and to be able to recommend more firmly this methodology for future studies? Please comment and add to the discussions.

The aim of this manuscript was not to validate the synthetic control (SC) method or compare to the more traditional interrupted time series (ITS) analysis as this has been previously described. Bruhn, *et al*. compared the performance of SC and ITS using hospitalization data from Brazil, Chile, Ecuador, Mexico, and the United States. They found that the impact estimated with the synthetic control and interrupted time series analysis was similar when there were no unexpected biases in the data, but impact could be masked when biases existed, for example an increase in pneumonia deaths related to the 2009 influenza pandemic. Based on this recommendation, we included results from an interrupted time series analysis in the supplementary material and added to the main manuscript:

Methods, sensitivity analysis section line 194-200 page 10: “**We performed four main types of sensitivity analysis (detailed in supporting information):** ……. 4) **estimating the impact of PCV using a different analytic method (interrupted time series model).**”

Results, sensitivity analysis section line 284 page 14:” **For all age groups with reductions in pneumonia mortality, the interrupted time series analysis predicted a greater reduction in deaths than the synthetic control model (Table S9)**.”

Discussion line 379 page 18: “The synthetic control approach is more suitable than interrupted time series analysis for assessment of PCV impact on hospitalisations,[10] and mortality.[4, 5] Interrupted time series does not account for changes in trends in the evaluation period not due to the intervention which may reduce the validity of the estimation of impact[38] **which was suggested by the over estimation of impact when the interrupted time series method was applied to these data**.”

7. On the same topic, you mention in your discussion that "Our selection of controls was limited to the data available, but results were robust to changes in controls on sensitivity analysis, suggesting that this approach was able to adequately control for sociodemographic and health system related changes." Could you explain this further? My question is: could the fact that your results did not change that much when you performed extensive sensitivity analyses, excluding big groups of causes from your control group, indicate the opposite, i.e. that these analyses were not being able to adequately control for sociodemographic and health system related changes?

One of the key assumptions for the synthetic control method is that the selected control disease and the outcome are influenced by the same/similar set of factors unrelated to PCV (e.g., changes in sociodemographic factors and healthcare system). If this assumption holds, excluding one of the selected control diseases should not change the estimated impact of PCV dramatically, as remaining selected control diseases should be able to adjust for these unrelated factors. If all covariates are removed (as in interrupted time series analysis (ITS)) and results remain the same, it shows that the synthetic control does not control for biases. When comparing the results obtained from the synthetic control approach to those obtained from the ITS analysis (now also included in the supporting information Table S9) it does show that the chosen analytical approach does indeed control for these changes in a manner the ITS does not, as the ITS estimates a 45-77% greater impact than the synthetic control approach. In the absence of a gold standard, we are unable to say if the synthetic approach controlled for sociodemographic and health system related changes perfectly, but we believe it is more accurate than the ITS estimates.

8. I did not understand what was done for the P code: "For the purpose of this study, all P codes used in children aged >1 month were grouped with non-P codes where possible, as previously described". When you say there was "a systematic misclassification of ICD codes among children aged <1 year into the P code groups", could have been pneumonia J12-J18 deaths among those coded with P codes? Please explain in a clearer format.

Detailed methods moved to supplement to allow for longer explanations, and the following example was added to clarify (paragraph 2 sensitivity analysis methods section, page 3 supporting information): “For the purpose of this study, all P codes used in children aged >1 month were grouped with non-P codes where possible, as previously described.[5] **For example, if a death was coded as anything between P35-P39 (Infections specific to the perinatal period) it was included in the control group that included the A and B chapters (Certain infectious and parasitic diseases) and deaths with P23 in any of the cause of death fields were included as a pneumonia death**.”

9. The percentage of deaths with a cause of death from ICD-10 R chapter indicated as cause of death (main, underlying or other cause of death) increased abruptly for children aged 1-11 months in 2006. What proportion of those were classified as cases or controls, pre and post vaccination? This needs to be further explained. What is the influence of this change in the results for the target age group? I do not think the exclusions of the R codes from the controls tackles this issue.

This coding change affects only the 1-11-month old group. The aim of excluding the R chapter in a sensitivity analysis was to assess if the ill-defined causes of death in this chapter influenced the final effect calculated. For example, if some pneumonia deaths coded as R (and had no other causes of deaths coded as J12-18) it would have biased the results towards a lower effect observed. We agree that this sensitivity analysis does not address the abrupt change in coding for the 1-11 months old group. One of the additional sensitivity analyses included after review, was the exclusion of 1999-2005 data, in essence excluding the period where the shift in coding practises occurred. This puts the start of the pre-vaccine period at the point where pneumonia mortality already started to reduce, and results in a calculated effect of zero, which may indicate no indirect effect of the vaccine in this age group. We’ve added Figure S2 to show percentage of deaths with R codes in any cause of death field in control and outcome groups. We’ve also added details around this sensitivity analysis to the discussion, see line 338 - 348 page 16.

10. It was not clear to me why you performed sensitivity analyses excluding the following years: 2001 (two-years from start of data), 2008 (one year prior to introduction) and 2015 and 2016 (reducing evaluation period by 2 years). What does the fact that these exclusions did not change the results tell us? Please comment and add to the text.

In hindsight, the decision on the years to exclude was somewhat arbitrary, with the aim to see if the results changed, but in a hit or miss approach. We’ve removed the analysis for 2001 and 2008, and rather excluded 1999-2005 (excluding the period in which pneumonia mortality increased, and when coding practices in the 1-11-month old age group changed, and 2009 where the influenza pandemic may have influenced the results.

11. You are analyzing this time-series several years after vaccine introduction. You suggest some possibilities for the lack of indirect effect found in older individuals. But what about serotype replacement? Could it be reducing the observed vaccine effects in younger individuals and contributing to the lack of effect in adults? A word on that would be welcomed.

Unfortunately, our analysis was not able to detect these changes due to the nonspecific end-point used. We included this as a limitation of the study in the final discussion paragraph, line 393 - 399 on page 18: “**The vaccine impact evaluated here included the reduction of PCV7 and PCV13 serotype-related deaths, potential increase in non-vaccine serotypes-related deaths (replacement), the direct effect on vaccinated children and the indirect effect on the non-vaccinated population and we were not able to distinguish these effects individually using this ecological approach. Shortening of the analysis period prior to the increase of non-vaccine serotypes in South Africa was not possible as the increase in non-vaccine serotypes was already signalled in invasive pneumococcal disease in 2010. [24]**”.

12. I think we can assume that the impact of vaccination could be greater with a greater vaccination coverage. Perhaps a word on this could be added to the discussion/conclusion.

Added to the discussion, line 307-311, page 15: “**This reduction in all-cause pneumonia mortality was observed against a backdrop where coverage of a third dose of PCV in South Africa was initially low with 62% in 2011, increased to 85% in 2014 and 2015, and decreased again to 77% in 2016.[7] Higher vaccine coverage in South Africa may lead to a larger reduction in pneumonia deaths, as higher levels of uptake of the vaccine was associated with larger declines in all-cause pneumonia hospitalizations[21] and pneumonia mortality [22] in Brazil.**”

-----------------------------------------------------------

**Reviewer #3:**

The study "Impact of the pneumococcal conjugate vaccine on pneumonia mortality in South Africa, 1999 through 2016" is aiming to fill a glaring gap in our understanding of PCV impact in Africa; that is the impact of PCV on (pneumonia) mortality. The study is well written and generally conducted well. The ecological analysis however, has to fully rely on a non-mechanistical machine learning like approach because of major changes in pneumonia mortality observed at baseline that likely have continued during the period of vaccination. I think the synthetic control methodology is the best approach to estimate impact in this situation but my main concern is that we will need to completely rely on it and hence I would like to see a number of additional sensitivity analyses to ensure the robustness of the approach.

1. The synthetic control is a powerful analysis and one of the very few ways to extract a signal off the noisy data at hand. But it comes with a number of caveats. These include: 1) covariate selection is not hypothesis driven 2) which in combination with the very detailed data at hand makes it prone to over fitting and 3) it is not necessarily clear that the relationship with pneumonia mortality would stay the same in the post vaccination era. The authors do a number of helpful sensitivity analysis but I think it would be good to also do the following: A Morris Method like approach where you re-run the analysis a couple of hundred time and for each run you randomly select only 2/3 of the available set of covariates

One of the sensitivity analysis currently included in the study removes the top three weighted controls (first the top 1, then the top 2 and lastly the top 3) from the model, Table S4. We therefore opted to not employ the Morris Method.

2. To evaluate the balance of making best use of potential signals and limiting the potential for over-fitting I would like to see the analyses re-done but on data in weekly as well as annual aggregation

We did consider running the analysis with weekly and annual data, but we do not believe that this would assess over-fitting. Reducing the data points by running a yearly analysis could rather lead to over fitting, and results in too few data points for the model to run. On the contrary, weekly aggregation will lead to noise which may obscure relationships between the outcome and controls. Overfitting is more likely when too few data points are used with too many covariates. Although there were 38 possible control groups, the model included on average 24 controls (range 14-32), along with the 11 monthly dummy covariates, resulting in around 35 covariates included in each model with 132 data points (11 years’ pre-intervention, monthly data) which reduces the concern for overfitting.

3. Further, some of the authors have used the method in another setting, Brazil, before. While relevant covariates may differ to SA, some may actually have a more engrained role. I suggest to
   a. report not only the ICD codes used in the control but reported on their contribution to the control

The top three controls with their inclusion probability (which translate to weight) has been added Table S3.

b. compare those to the ICD codes that contributed to the Brazil analysis and

The top three controls included in the synthetic control model looking at the impact of PCV on pneumonia mortality in Brazil in the 3 months - 5 years old were all non-PCV related deaths, Intestinal infectious diseases (A00-A09) and Ill-defined and unknown causes of mortality (R95-R99). In our analysis, the R chapter was the third top control for the 1-11-month group but no other similarities were observed in the 1-4-year group.

c. rerun the analysis but only include the covariates that were deemed relevant for the Brazil analysis to see whether the control can still be fit.

We opt to not include the suggested additional analysis. This study’s objective was not to apply the same model that was used in other settings, where changes in sociodemographic factors and healthcare systems are different from South Africa. A study which includes data from multiple countries may be more suitable for comparing control groups between settings. The study by de Oliveira *et al.* which included ten countries may be of interest.

4. On page 6 the grouping of age groups into vaccine eligible needs a bit more nuancing. E.g the 1m-4y old age group has only been completely age eligible for vaccination from 2013.

Eligibility for vaccination were based on the final year of data included in this study. Children younger than 4 years in 2016 (born 2012-2016) could all have received the vaccine, where as those aged 5-7 (born 2009-2011) could have possibly received the vaccine, and anyone older than 8 years (born prior to 2009 when PCV7 was introduced) were not eligible to receive the vaccine. To clarify this in the text we’ve added (now moved to detailed methods in supporting information, page 2): “Individual-level mortality data were grouped according to ICD-10 chapter, month of death, and age group. Age strata in children were selected to assess the impact of PCV, **based on year of birth,** on those eligible for routine vaccination (1 month to 4 years), those who could possibly have been vaccinated in catch-up campaigns (**born before February 2009**) or routine vaccine (**born February 2009 – 2011**, 5-7 years) and those not eligible for vaccination (born before 2009, 8-18 years) **in 2016 the final year of data included in the analysis**.”

5. Similarly, Table 1 is quite puzzling in that for the age groups 1-4y and 5-7y there is basically the same annual number of deaths prevented from the start of PCV vaccination. One would rather have expected that this would gradually build up as such cohorts get vaccinated and herd immunity kicks in. Could the authors compare this with the timelines of effects on IPD to get a better understanding whether this makes sense or not?

Based on a previous study investigating the effect of PCV on invasive pneumococcal disease, the reduction of IPD incidence was evident from the first year of vaccine implementation. We’ve added to the discussion line 302-306, page 15: “Reductions in the number of deaths were observed from the first year of PCV7 introduction in 2009 in children aged 7 years and younger, and only from 2012 in children aged 8-18 years. **This corresponds to the reduction of invasive pneumococcal disease incidence already observed from 2009, especially in children younger than 2 years.[8]**”

6. The authors assess the impact of PCV. However, PCV7 was replaced with PCV13 in 2011 and in principle their analysis should allow to estimate the additional effect of PCV13 on top of PCV13 which would be interesting to try and tease out. It looks like there may be a signal in the <12m olds but it may be more difficult in the older children. As effects would overlap with the gradual build up of herd immunity.

We are unable to measure this using this study as serotype-specific data is not available, and any speculation would be circumstantial. We’ve added to the limitations paragraph (line 393-397 page 18): “**The vaccine impact evaluated here includes the reduction of PCV7 and PCV13 serotype-related deaths, potential increase in non-vaccine serotypes-related deaths (replacement), the direct effect on vaccinated children and the indirect effect on the non-vaccinated population and we are not able to distinguish these effects individually using this ecological study**.”

-----------------------------------------------------------

We hope that you find our responses and additions to the manuscript satisfactory. Please do not hesitate to contact me if you require any additional information.

Kind regards

**Ms. Jackie Kleynhans**

Epidemiologist

Centre for Respiratory Diseases and Meningitis (CRDM)

National Institute for Communicable Diseases (NICD)

1 Modderfontein Road

Sandringham

2192

Gauteng, South Africa

Tel: +27 (0)11 555 0383

Email: [jackiel@nicd.ac.za](mailto:jackiel@nicd.ac.za)
