# Supplementary material for: Estimated impact of the pneumococcal conjugate vaccine on pneumonia mortality in South Africa, 1999 through 2016: An ecological modelling study
Source: PLoS Med. 2021 Feb 16;18(2):e1003537. doi: 10.1371/journal.pmed.1003537 (PMC7924778; doi:10.1371/journal.pmed.1003537)
Supplement: Attachment — Submitted filename: Responses Kleynhans PCV impact mortality RSA Subm 3.docx [file pmed.1003537.s018.docx]

4 January 2021

**Re: Response to comments on manuscript** **PMEDICINE-D-20-04020R2**

Please find herewith the submission of the revised manuscript “**Estimated impact of the pneumococcal conjugate vaccine on pneumonia mortality in South Africa, 1999 through 2016: an ecological modelling study**”, and below detailed responses to the editor and reviewer’s comments. Original comments shown in black font and responses in green.

**Requests from the editors:**

1. Thank you for including your analysis plan, please make this a standalone supplementary file.

We removed the analysis plan from the main supplement file and added it as S3.

2. Please ensure that the study is reported according to the RECORD guideline, and include the completed RECORD checklist as Supporting Information. Please add the following statement, or similar, to the Methods: "This study is reported as per the REporting of studies Conducted using Observational Routinely-collected health Data (RECORD) Statement (S1 Checklist)." The RECORD guideline can be found here: <https://www.equator-network.org/reporting-guidelines/record/>. When completing the checklist, please use section and paragraph numbers, rather than page numbers.

We completed the RECORD checklist and added it as S1. The required statement was added to methods line 175-6.

3. As this is a modelling study based on observational data, please edit causal language throughout the manuscript, beginning with the title, which can be edited to “Estimated impact…”, as well as editing words such as “effect” at lines 51-2, 242-3, 341-2, and “prevented" at line 352. Please do check for any language we may have missed.

We have updated the language to be more appropriate for an observational study. Changes made:

- Title: “**Estimated** impact of the pneumococcal conjugate vaccine on pneumonia mortality in South Africa, 1999 through 2016: an ecological modelling study”
- Abstract line 42-9: “Compared to number of deaths expected, we ~~observed~~ **estimated** a 33% (95% credible interval (CrI) 26%-43%), 23% (95%CrI 17%-29%), 25% (95%CrI 19%-32%) and 23% (95%CrI 11%-32%) reduction in pneumonia mortality in children aged 1-11 months, 1-4 years, 5-7 years and 8-18 years in 2012-2016, respectively. In total, ~~there were~~ **an estimated** 18 422 (95%CrI 12 388-26 978) pneumonia-related deaths were prevented from 2009-2016 in children aged <19 years. No declines were **estimated** ~~observed~~ among adults following PCV introduction. This study was mainly limited by coding errors in original data ~~(although corrected to the best of our ability)~~ that could have led to a lower impact estimate.”
- Abstract line 52-3: “~~We observed~~ **The model predicted** an effect of PCV in age groups who were eligible for vaccination (1 months-4 years), and an indirect effect in those too old (8-18 years) to be vaccinated.”
- Author summary line 76-7: “**We estimated that** pneumonia mortality reduced between 23% - 33% in children younger than 19 years, with **an estimated** ~~more than~~ 18,000 deaths prevented between 2009-2016.”
- Results line 210-2: “In total, ~~there were~~ **an estimated** 18 422 (95% CrI 13 12 388-26 978) pneumonia-related deaths **were** prevented by PCV from 2009-2016 in children <19 years.”
- Results Table 1 title line 214: “Number of **estimated** deaths prevented in children <19 years since PCV introduction, South Africa, 2009-2016.”
- Discussion line 242: “We ~~observed~~ **estimated** a substantial reduction in all-cause pneumonia mortality in children aged 1 month to <19 years in South Africa after the introduction of PCV in 2009.”
- Discussion line 247-8: “We also ~~observed~~ **estimated** a 23% reduction in mortality in children aged 8-18 years who were too old to be eligible for vaccination. The **estimated** effect of the vaccine in children eligible for vaccination was observed from 2009 onwards, with the indirect effect of the vaccine in those not eligible for vaccination evident from three years after the vaccine introduction in 2009.”
- Discussion line 260: “This **estimated** reduction in all-cause pneumonia mortality was observed against a backdrop where coverage of a third dose of PCV in South Africa was initially low with 62% in 2011, increased to 85% in 2014 and 2015, and decreased again to 77% in 2016 [7].”
- Discussion line 275: “On sensitivity analysis by excluding the R chapter, a 19% reduction of pneumonia mortality was ~~observed~~ **estimated** in the 19-39-year age group, where the highest burden of HIV-infection and HIV-related mortality lies in South Africa [21].”
- Conclusion line 366-9: “**This study estimated that** the introduction of PCV in South Africa has resulted in a notable reduction of all-cause pneumonia mortality in children eligible for vaccination, as well as those not eligible for vaccination, which is in-line with previous projections. PCV **was estimated to** have prevented approximately 2 300 deaths annually from 2009-2016 in children <19 years.”

4. At line 47-78, please remove “(although corrected to the best of our ability)”, and add a point to note the possible influence of unmeasured confounders.

This study was mainly limited by coding errors in original data ~~(although corrected to the best of our ability)~~ that could have led to a lower impact estimate, **and unmeasured factors could also have confounded estimates**.

5. Please add “This study found that…” or similar language in the Abstract conclusions and the Discussion conclusion.

We updated the language as follows:

- Abstract, conclusion, line 51: “**This study found that** the introduction of PCV was associated with substantial reduction in all-cause pneumonia deaths in children aged 1 month to <19 years. ~~We observed~~ **The model predicted** an effect of PCV in age groups who were eligible for vaccination (1 months-4 years), and an indirect effect in those too old (8-18 years) to be vaccinated.”
- Conclusion line 363-7: “**This study estimated that** the introduction of PCV in South Africa has resulted in a notable reduction of all-cause pneumonia mortality in children eligible for vaccination, as well as those not eligible for vaccination, which is in-line with previous projections.”

6. Line 58, “pneumonia”

Line 59 updated: “The bacterium, *Streptococcus pneumonia****e***, causes a third of childhood pneumonia deaths in Sub-Sahara Africa.”

7. The second section of the Author summary could include a description of the study design (using national death registration data in South Africa from 1999-2016 to assess the impact of PCV introduction on all-cause pneumonia mortality) before describing the synthetic control.

Added to line 68-70 in author summary: “**We performed an ecological modelling study using national death registration data in South Africa from 1999-2016 to assess the impact of PCV introduction on all-cause pneumonia mortality**”

8. Please move the reference callouts to before the punctuation.

All citations moved to after punctuation marks. Please note this was not done as part of tracked changes.

9. In the Discussion Conclusion, please temper the claim of primacy, e.g., “To our knowledge, these are the first national-level data…”

Added to conclusion line 366: “**To our knowledge,** these are the first national-level data from an African country showing the impact of PCV on pneumonia mortality.”

10. The Contributors, Declaration of interest, Funding statements can go from the end of the text and into the submission metadata.

These sections were removed from the main manuscript.

**Comments from Reviewers:**

Reviewer #1: Thank you to the authors for addressing my previous comments well. I have no further issues to raise.

Reviewer #2: The manuscript has improved substantially after the reviewer's comments. The authors have replied satisfactorily to all the issues that had been raised.

Reviewer #3: The authors have addressed some of my comments but have decided to not run any of the suggested sensitivity analyses. As a result, I remain a bit concerned about the robustness of the inference. In the end you find correlations in any large enough data set which doesn't necessarily mean that those correlations are predictive of future changes. In more detail:

- While I think the Morris method is more robust I can see that the author's approach to exclude the top three controls is not too dissimilar, so that's okay I guess
- The authors argue their case to not use different data aggregation. If the inference indeed is only valid if run on months that still does tell the reader something about the robustness of the inference and should hence be included as a discussion point I think.

We added to the discussion, limitations line 359-61: “**We only used monthly aggregated data and did not assess the model using weekly or yearly aggregated data.** **Different aggregation could possibly affect estimates.**”

- The important covariates to model the outcome were very different in this analysis compared to a similar one done for Brazil. While it is entirely possible that this is a true difference it also should be included as a discussion point. Similarity of important covariates would have been somewhat reassuring that there is some structure in what predictors for pneumococcal disease changes are. Similarly it would have been reassuring to see that a similar model to the Brazilian one would have led to similar results; though I agree that absence of such doesn't necessarily invalidate the results in this study

We added the following to the discussion line 314 - 325: “**Using a similar methodological approach** in Brazil, Schuck-Paim *et al*. found that on a national level there were no pneumonia mortality reductions attributable to PCV in children aged <5 years (on a background of substantial reductions due to economic improvements). PCV likely contributed to a reduction in pneumonia mortality of between 16% and 24% in Brazilian municipalities with a high percentage of extreme childhood poverty and mothers without primary education [4]. **The South African model used different controls in the synthetic model as applied in Brazil. The top three controls included in the synthetic control model in Brazil in the 3 months - 5 years old were all non-PCV related deaths, Intestinal infectious diseases (A00-A09) and Ill-defined and unknown causes of mortality (R95-R99). In our analysis, the R chapter was the third top control for the 1-11-month group but no other similarities were observed in the 1-4-year group. This highlights how setting-specific trends may influence the utility of certain control groups when using the synthetic control approach**.”

-----------------------------------------------------------

We hope that you find our responses and additions to the manuscript satisfactory. Please do not hesitate to contact me if you require any additional information.

Kind regards

**Ms. Jackie Kleynhans**

Epidemiologist

Centre for Respiratory Diseases and Meningitis (CRDM)

National Institute for Communicable Diseases (NICD)

1 Modderfontein Road

Sandringham

2192

Gauteng, South Africa

Tel: +27 (0)11 555 0383

Email: [jackiel@nicd.ac.za](mailto:jackiel@nicd.ac.za)
